# Frontal and parietal theta burst TMS impairs working memory for visual-spatial conjunctions

**DOI:** 10.1016/j.brs.2012.03.001

**Published:** 2013-03

**Authors:** Helen M. Morgan, Margaret C. Jackson, Martijn G. van Koningsbruggen, Kimron L. Shapiro, David E.J. Linden

**Affiliations:** a Wolfson Centre for Clinical and Cognitive Neuroscience and Wales Institute of Cognitive Neuroscience, School of Psychology, Bangor University, Penrallt Road, Gwynedd, LL57 2AS, UK; b MRC Centre for Neuropsychiatric Genetics & Genomics, Institute of Psychological Medicine and Clinical Neurosciences, School of Medicine, Cardiff University, UK; c School of Psychology, Cardiff University, UK

**Keywords:** Working memory, Transcranial magnetic stimulation, Conjunction, Colour, Orientation

## Abstract

In tasks that selectively probe visual or spatial working memory (WM) frontal and posterior cortical areas show a segregation, with dorsal areas preferentially involved in spatial (e.g. location) WM and ventral areas in visual (e.g. object identity) WM. In a previous fMRI study [1], we showed that right parietal cortex (PC) was more active during WM for orientation, whereas left inferior frontal gyrus (IFG) was more active during colour WM. During WM for colour-orientation conjunctions, activity in these areas was intermediate to the level of activity for the single task preferred and non-preferred information. To examine whether these specialised areas play a critical role in coordinating visual and spatial WM to perform a conjunction task, we used theta burst transcranial magnetic stimulation (TMS) to induce a functional deficit. Compared to sham stimulation, TMS to right PC or left IFG selectively impaired WM for conjunctions but not single features. This is consistent with findings from visual search paradigms, in which frontal and parietal TMS selectively affects search for conjunctions compared to single features, and with combined TMS and functional imaging work suggesting that parietal and frontal regions are functionally coupled in tasks requiring integration of visual and spatial information. Our results thus elucidate mechanisms by which the brain coordinates spatially segregated processing streams and have implications beyond the field of working memory.

## Introduction

Working memory (WM) is a system for temporary maintenance and manipulation of verbal and visuo-spatial information [2]. In the visual domain, it has been proposed that WM is separated into separate visual (e.g. object identity) and spatial (e.g. location) processing streams, corresponding to the ventral (visual) and dorsal (spatial) segregation of perceptual processing [3,4]. This separation of visuo-spatial WM has been demonstrated behaviourally using dual task methodology, in which two tasks are performed simultaneously. Interference between the two tasks is lower when one task requires visual WM and the other task requires spatial WM, compared to when both tasks share similar processes [5–7].

The dorsal/ventral segregation of WM is further supported by brain imaging studies, which show that WM for spatial information involves superior parietal and prefrontal areas, whereas inferior prefrontal areas are preferentially involved in WM for non-spatial information [8]. In particular, inferior frontal gyrus (IFG), and parietal cortex consistently show domain-specific activity during WM for visual-spatial information. Several studies have shown that WM maintenance of spatial information, such as location or orientation, is associated with increased activity in parietal cortex [9–15], compared to maintenance of visual object information. This likely reflects the role of the parietal cortex in spatial processing [16]. By contrast, WM maintenance of visual information, such as identity or colour, produces increased activity in IFG, either bilaterally [9,11,12] or predominantly on the left [10,14,15,17]. IFG also seems to be involved in other cognitive operations on non-spatial visual object representations, such as encoding faces [18,19] or attending to colours [20,21]. In other work we have used fMRI-guided ERP source modelling to show spatiotemporal dissociations between visual and spatial processing streams during WM, with domain-specific activity in IFG during encoding and retrieval and in parietal cortex during the WM delay [22].

Other accounts of anatomical dissociations of WM-related activity have focussed on different task types or processes. It has been proposed that dorsolateral prefrontal cortex (DLPFC) is involved in active monitoring and manipulation of information, whereas ventrolateral prefrontal cortex (VLPFC) is involved in retrieval of information from posterior areas [23,24]. However, domain- and process-based accounts of WM architecture are not mutually exclusive [25]. Behavioural work has shown that colours and orientations can be simultaneously manipulated in WM with no impairment in performance relative to manipulation of a single feature, whereas dual task performance was impaired when both tasks involved spatial information [26]. This suggests that active manipulation of WM processes at least partly depends on domain-specific representations. Indeed, fMRI studies have shown that certain dorsal and ventral frontal and parietal areas are recruited in an information-specific manner independent of the type of WM process, whereas other regions appear to be specialized according to the executive demands of the task [14,17].

**Figure 1:**
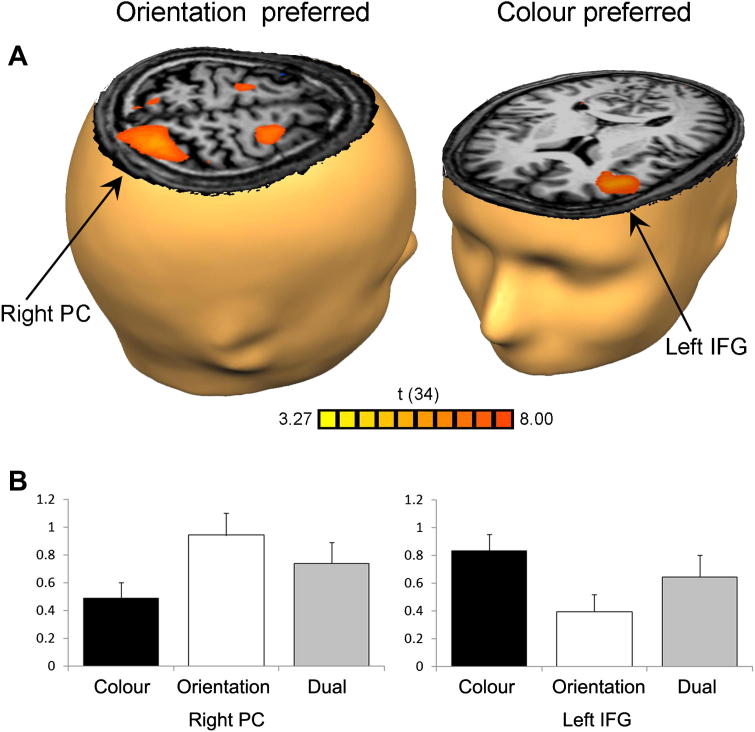
Results from the fMRI study [1]. (A) Group statistical maps of orientation minus colour (orientation-preferred) and colour minus orientation (colour-preferred) contrasts (FDR < 0.05), showing the right PC and left IFG TMS stimulation sites. (B) Plots showing beta values from these areas during colour, orientation, and dual trials, with bars showing the standard error of the mean.

An important function of normal human cognition is the ability to integrate information. But the way in which visual and spatial information is integrated in WM is less well understood. Studies using fMRI have shown that WM for visual-spatial conjunctions recruits visual- and spatial-preferred regions, but the level of activity in these regions is intermediate to the activity produced by the preferred and non-preferred single task information [10,15]. In recent work we used fMRI to examine WM for visual and spatial information and visual-spatial conjunctions [1]. In a delayed matching-to-sample paradigm modelled after Mohr and Linden [26], participants were instructed to mentally manipulate the colours, orientations, or both colours and orientations (dual task) of two briefly presented sample stimuli, and then indicate whether or not a subsequent test stimulus was the intermediate colour blend and/or orientation of the previous samples. Colour-preferred activity was observed in left IFG and right occipital cortex, whereas orientation-preferred activity was found in right parietal cortex, bilateral superior frontal sulcus, and left temporal cortex (see Fig. 1). Consistent with the results of previous studies, dual task activity in each of these areas was intermediate between the activity produced by the preferred and non-preferred task. Therefore, it remains unclear whether activity in domain-preferred regions plays a critical role in WM for visual-spatial conjunctions, or whether this activity is simply an epiphenomenon.

The task-critical role of regional neural activity during WM can be examined using transcranial magnetic stimulation (TMS). This technique induces brief functional deficits, allowing the investigation of causal links between neural activity and cognitive performance [27–29]. The results of TMS studies into visuo-spatial WM are largely consistent with fMRI work, showing a dorsal/spatial and ventral/visual organisation of WM. Kessels et al. [30] showed that repetitive TMS (rTMS) over right parietal cortex during the retention interval interferes with WM for spatial locations, whereas rTMS over left parietal cortex did not affect spatial WM performance. Oliveri et al. [31] found that bilateral TMS over parietal cortex or superior frontal gyrus selectively disrupted WM for spatial locations, whereas bilateral TMS over middle temporal regions disrupted WM for abstract patterns. Mottaghy et al. [32] showed that 10 min of 1 Hz rTMS over dorsomedial prefrontal cortex (PFC) impaired performance on a subsequent spatial location WM task, whereas 1 Hz rTMS over ventral PFC impaired subsequent face-recognition performance.

However, TMS studies of WM have not examined WM for conjunctions of visuo-spatial information. Thus the role of domain-specific areas in conjunction tasks involving spatial and identity information, relative to their role in the respective single feature tasks, is not clear and is the purpose of the present study. We examined how TMS over visual- and spatial-preferred brain regions influences WM for visual-spatial conjunctions, as well as single features. That is, do these domain-specific regions play a critical role in the coordination of spatially separate processing streams? Based on the regions of colour- and angle-preferred activity found in our previous fMRI study, we selected left IFG and right PC as TMS stimulation sites. These sites were chosen because they showed the highest mean beta values in the fMRI study, they correspond well to spatial- and object-preferred regions identified in previous work [9,10,15], and they have been found to be content-specific for both maintenance and manipulation of WM contents [14].

We applied theta burst stimulation, which has been shown to induce a long-lasting deactivation of the underlying cortex. For example, Huang et al. [33] found that 40 s of continuous theta burst stimulation over motor cortex led to a reduction in the amplitude of the motor evoked potential, which lasted for 60 min. This inhibitory effect was not observed immediately, but rather emerged slowly during the first 10 min post-stimulation. We chose this offline TMS method because it would not be influenced by possible differences in the timing of activity in colour- and orientation-preferred regions. Theta burst TMS over prefrontal and parietal regions has been found to modify performance in several other cognitive tasks. Theta burst stimulation of prefrontal cortex has been shown to impair task preparation [34], prospective memory [35], conjunction search [36], and behavioural updating [37], and reduce impulsivity in decision making [38]. Theta burst stimulation of inferior parietal cortex has been found to impair sequence learning [39].

Because the effects of theta burst stimulation take time to build up, we examined the effect of TMS stimulation compared to sham TMS (baseline) over three separate time intervals: before TMS, immediately after TMS (post-TMS 1), and 11–22 min following TMS (post-TMS 2). The before TMS measurement was to confirm that task performance before administering TMS did not vary according to the type of session (sham TMS, left IFG TMS, and right PC TMS), and that performance on all tasks was above chance without TMS. Based on previous work [33], we expected to find interference from TMS stimulation during the second time interval after application of theta burst TMS.

## Materials and methods

### Participants

Twenty neurologically healthy volunteers (mean age 25 years; 10 females) from the student and community panels of the School of Psychology, Bangor University volunteered to take part in both the fMRI study [1] and the current TMS study in return for payment. Of these, 2 participants were excluded due to excessive head movement inside the fMRI scanner, yielding a sample size of 18 for the fMRI measurements (see Ref. [1]). Of the original 20 volunteers, 5 participants withdrew from the TMS part of the study, and one further participant was excluded due to chance performance at baseline and excessive nervousness. These were replaced with an additional 3 female volunteers (mean age 25 years), yielding a sample size of 17 for the current TMS experiment.

### Stimuli and procedure

**Figure 2:**
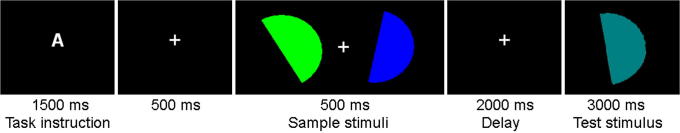
An example of the sequence of events in a typical trial. An instruction letter indicated which task to perform. Participants had to manipulate the colours, orientation angles, or both colours and angles (dual task) of the sample stimuli to determine whether the test stimulus matched or mismatched the average colour and/or angle of the two samples.

The experiment required manipulation of colours, orientations, or both colours and orientations in WM. Fig. 2 shows an example of the trial sequence. Stimuli were presented on a 17″ TFT monitor and participants responded using keys “A” and “L” on the computer keyboard. The stimuli were coloured semi-circles (visual angle = 2.2° × 4.1°) on a black background. The orientation angles of the two sample stimuli differed by a rotation of 60°. In the “match” condition of the angle task, the orientation angle of the test stimulus differed from each sample stimulus by a rotation of 30° (i.e. the test stimulus was the average). In the “mismatch” condition, the orientation angle of the test stimulus differed from the average angle by 20° (50%) or 30° (50%). Colours were defined in Hue Saturation Value (HSV) colour space, in which the hue is represented by 0-360°. The two sample stimuli differed in hue by 60°, and the hue of the test stimulus in the “match” condition differed from each sample by 30° (i.e. the test stimulus was the average hue). In the “mismatch” condition, the hue of the test stimulus differed from the average hue by either 50° (50%) or 30° (50%). The colours of the sample and test stimuli were matched in luminance.

Each trial began with a central instruction letter, indicating which task to perform. “A” indicated the orientation (angle) task, “C” indicated the colour task, and “D” indicated the dual task (i.e. the combination of both orientation and colour tasks). A white fixation cross appeared for 500 ms, then two sample stimuli with different colours and orientations appeared for 500 ms on the left and right of the fixation cross (distance from fixation = 3.8°). There was a 2000 ms delay in which only the fixation cross was present. Then a test stimulus appeared in the centre of the screen for 3000 ms, during which participants had to indicate with a left- or right-hand button-press whether or not the test stimulus was the average of the two sample stimuli in terms of colour, orientation, or both colour and orientation. This was followed by a feedback display for 1000 ms, in which the fixation cross turned green for a correct response, red for an incorrect response, and grey if no response was made during the 3000 ms presentation of the test stimulus. The assignment of match and mismatch to the left and right response buttons was counterbalanced across participants. For each task, the test stimulus matched the average of the two samples on one-third of trials and mismatched on two-thirds of trials. There was a 2 s inter-trial interval, which contained only the fixation cross. The experiment was divided into three separate 11 min blocks of 63 trials (pre-TMS, post-TMS 1 and post-TMS 2), with 21 trials per task. The order of conditions within each block was randomised. A short practice was given at the beginning of each experimental session.

Participants completed three TMS sessions on separate days: sham TMS (baseline), left inferior frontal gyrus (IFG) TMS, and right parietal cortex (PC) TMS. The order of the TMS sessions was counterbalanced across participants and the sessions were separated by at least a week.

### TMS

Theta burst rTMS was delivered using a handheld figure of eight coil with an external loop diameter of 8 cm connected to a Magstim Super Rapid system (Magstim, Whitland, UK). Active motor threshold (aMT) was obtained for each participant by applying single TMS pulses over the hand area and determining the minimum stimulation intensity necessary to elicit a finger twitch during voluntary contraction of the hand muscles. Mean aMT was 61.5% (standard deviation = 6.3) of stimulator output. Based on the fMRI results [1] (see Fig. 1), we selected left IFG (talairach: *x* = −48, *y* = 32, *z* = 12) and right PC (talairach: *x* = 14, *y* = −58, *z* = 53) as the stimulation sites. Sham stimulation was applied over left posterior PC (talairach: *x* = −38, *y* = −71, *z* = 5). Talairach coordinates from the group fMRI analysis were used to locate the stimulation sites in each individual by using stereotactic registration to each individual's structural MRI (BrainSight, Magstim, Whitland, UK). Note that, although neuronavigation based on fMRI activity foci for individual participants has been shown to produce the best functional accuracy of TMS, neuronavigation based on group Talairach coordinates has relatively good functional accuracy provided that a sufficiently large sample size (*N* > 13) is used [40].

In the right PC session, the coil was placed tangentially to the scalp with the handle pointing backward and leftward at a 45° angle from the mid-sagittal line. In the left IFG session the coil was placed tangentially to the scalp with the handle pointing forward and downward. In the sham (baseline) session the coil was placed perpendicular to the scalp at a 45° angle from the mid-sagittal line. Participants first completed the pre-TMS block of WM trials, then a 40 s train of theta burst TMS was administered at 80% of aMT. This consisted of three pulses at 50 Hz, repeated at intervals of 200 ms (600 pulses in total [33];). Immediately afterwards, participants completed post-TMS WM blocks 1 and 2. No adverse reactions to theta burst TMS were reported.

## Results

**Figure 3:**
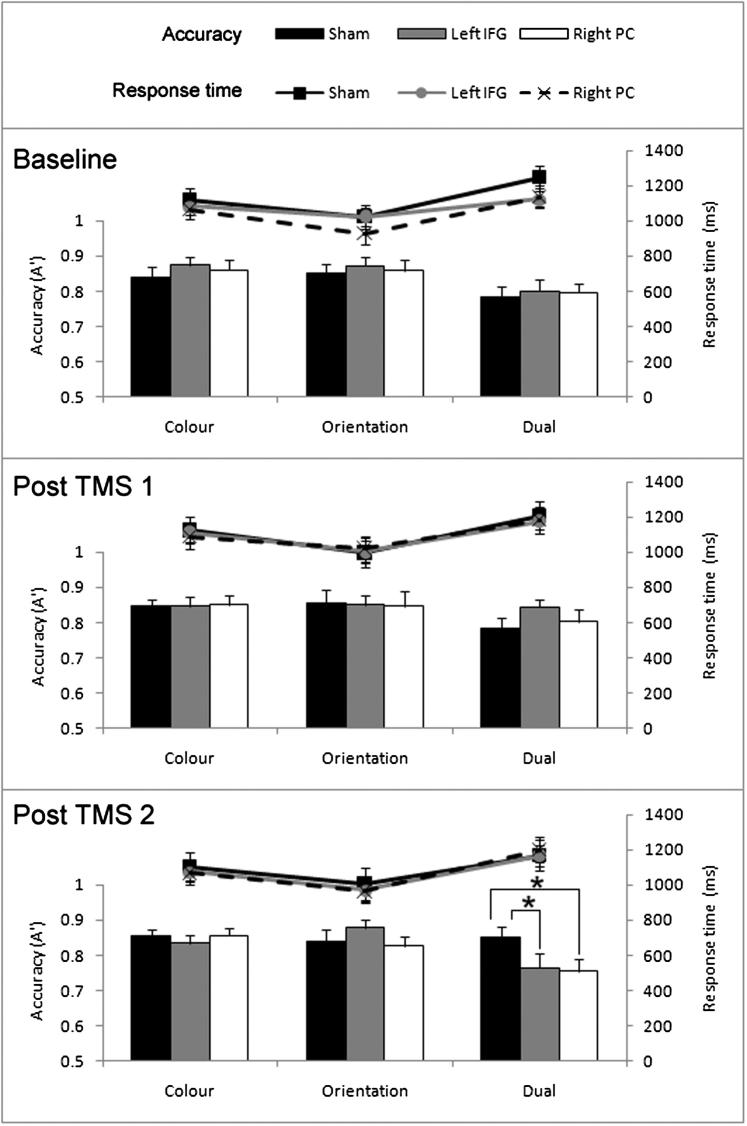
Mean accuracy (A prime) and response time in each task during the pre-TMS (baseline), post-TMS 1, and post-TMS 2 blocks for the sham, left IFG, and right PC TMS sessions. Error bars show the standard error of the mean. * indicates significant (*P* < 0.05) differences between conditions.

Mean response times and accuracy are shown in Fig. 3. Other work using rTMS has shown considerable practice effects in sham TMS conditions, and these practice effects were larger in a more demanding task requiring mental imagery compared to visual colour or angle discrimination tasks [41]. Therefore, to avoid the confound of practice effects, accuracy (A′)^1^ and response time (RT) data for each time interval (pre-TMS, post-TMS 1, and post-TMS 2) were analysed separately using 3 × 3 repeated-measures ANOVAs with the factors TMS session (baseline – sham TMS, left IFG TMS, and right PC TMS) and task (colour, orientation, and dual). Significant effects were followed up by Bonferroni-corrected post-hoc tests. Note that the pre-TMS time interval was included to ensure that performance without TMS did not vary across the three sessions.

### Pre-TMS

The accuracy analysis found a main effect of task, *F* (2,32) = 5.5, *P* = 0.009, with significantly lower accuracy in the dual task compared to the colour task (*P* < 0.05) and the orientation task (*P* = 0.02). As expected, there was no main effect of TMS session prior to administering TMS, *F* (2,32) = 0.6, ns, and no interaction, *F* (4,64) = 0.1, ns. The RT analysis also found a main effect of task, *F* (2,32) = 21.3, *P* < 0.001. RTs were slower in the dual task compared to the colour task (*P* = 0.03) and the orientation task (*P* < 0.001). RTs were also slower in the colour task compared to the orientation task (*P* = 0.02). As expected, there was no main effect of TMS session, *F* (2,32) = 2.3, ns, and no interaction, *F* (4,64) = 2.0, ns. The lack of an effect of TMS session in the accuracy and RT analyses confirms that participants performed consistently across the three TMS sessions.

### Post-TMS 1

Analysis of accuracy found no main effects of task, *F* (2,32) = 1.7, ns, or TMS session, *F* (2,32) = 0.5, ns, and no interaction, *F* (4,64) = 0.6, ns. The RT analysis showed a main effect of task, *F* (2,32) = 14.8, *P* < 0.001, with slower RTs in the dual task compared to the colour task (*P* = 0.047) and the orientation task (*P* < 0.001), and slower RTs in the colour task compared to the orientation task (*P* = 0.03). There was no main effect of TMS session, *F* (2,32) = 0.05, ns, and no interaction, *F* (4,64) = 0.7, ns.

### Post-TMS 2

The accuracy analysis revealed a main effect of task, *F* (2,32) = 4.4, *P* = 0.02, and no main effect of TMS session, *F* (2,32) = 2.1, ns. Of most interest, there was a significant interaction, *F* (4,64) = 2.9, *P* = 0.03 (see Fig. 3). TMS session had no effect on performance of the colour task, *F* (2,32) = 0.5, ns, or the orientation task, *F* (2,32) = 1.5, ns. However, there was a significant effect of TMS session on dual task performance, *F* (2,32) = 4.4, *P* = 0.02. Dual task accuracy was significantly lower after TMS over left IFG (*P* = 0.03) and right PC (*P* = 0.02) compared to sham TMS (Bonferroni-corrected). The RT analysis found a main effect of task, *F* (2,32) = 24.4, *p* < 0.001. RTs were slower in the dual task compared to the colour task (*P* = 0.003) and the orientation task (*P* < 0.001), and slower in the colour task compared to the orientation task (*P* = 0.02). There was no main effect of TMS session, *F* (2,32) = 0.1, ns, and no interaction, *F* (4,64) = 0.8, ns.

## Discussion

We investigated WM for colours, orientations, and colour-orientation conjunctions following theta burst rTMS over colour- and orientation-preferred regions compared to sham stimulation. The single tasks were balanced for accuracy, but participants were slower to respond in the colour compared to the orientation task. Theta burst rTMS over left IFG and right PC led to impaired WM performance relative to baseline (sham TMS) when the task involved manipulation of colour and orientation conjunctions (dual task), but not when a single feature was manipulated. The effects of theta burst TMS did not emerge until the second post-TMS block, 11–22 min after rTMS was administered. This is consistent with Huang et al.’s study [33], in which the inhibitory effect of theta burst TMS on motor evoked potentials was not observed immediately, but rather built up slowly during the first 10 min post-stimulation. This suggests that colour-preferred (IFG) and orientation-preferred (right PC) regions play a role in integrating the two types of information in WM.

Note that the dual task required WM manipulation of two features, whereas the single feature tasks only required one type of information to be manipulated. However, it is unlikely that the impaired dual task performance following rTMS is due to increased demands on WM resources in the dual task. This is because increasing WM load is typically associated with increased BOLD signal in prefrontal and parietal areas [42–45]. By contrast, our previous fMRI results [1] show intermediate activation in left IFG and right PC during the dual task. That is, dual task activity in each region was less than the activity produced by the preferred single feature task and greater than the activity produced by the non-preferred task [1], indicating that the IFG and PC activity found in this paradigm does not scale with task difficulty. Furthermore, other work has shown that WM capacity for simple object features, such as colours, is around 4 items [46–50], which suggests that the requirements of the conjunction task in the current study are within normal capacity limits.

It has been suggested that prefrontal regions play a role in monitoring and manipulating information in WM [51,52], Prefrontal cortex activity during WM often shows a dorsal-spatial/ventral-visual preference for the type of stimulus material [9–15,17], suggesting that the posterior ventrodorsal dissociation of perceptual processing [3] continues into prefrontal areas involved in WM [4]. However, prefrontal cortex is also involved in a wide range of cognitive functions; in particular, lateral prefrontal regions are involved in cognitive coordination in dual task situations [53–57]. This could explain why conjunction tasks, which require cognitive coordination, are selectively impaired by prefrontal TMS.

Although parietal lobe is activated more by WM tasks involving spatial compared to visual information [9–15], this region is thought to play an important role in WM storage of both visual and spatial information [42,45], and superior parietal lobe is activated more by WM tasks which have greater executive control requirements [8]. The lateral intraparietal area of monkeys contains both shape- and location-selective neurons [58,59], and recent fMRI work has shown that visual object information is represented in parietal areas [60]. The representation of multiple aspects of objects may allow the parietal lobe to integrate different types of information in WM. Indeed, neuropsychological evidence supports the idea that parietal lobe is critical for forming integrated representations. Patients with Balint's syndrome, with bilateral parietal damage, show binding problems on tasks requiring conjunctions of simple visual features [61,62], and patients with unilateral parietal lesions are selectively impaired on conjunction search in their contralesional visual field [63]. It is possible that feature integration in WM depends on the parietal spatial attention network [64]. This might explain why TMS in the current study selectively affected the conjunction task but not the single feature tasks.

However, these explanations of parietal and prefrontal involvement in conjunction tasks do not explain the intermediate fMRI activity in right PC and left IFG that we observed for conjunctions compared to single features [1]. Indeed, WM for conjunctions may specifically involve the left parietal cortex [1,65], rather than the right parietal region that was targeted by TMS in the current study, and which seems to reflect domain-specific activity. It is possible that domain-specific processing in one pathway (e.g. colour-ventral) is inhibited by processing in the other pathway (e.g. orientation-dorsal), and vice versa [10]. Indeed, other work has shown that parietal and frontal regions are functionally coupled in a task requiring visuo-spatial judgements [66]. This would lead to the intermediate dual task activation observed in the fMRI results. rTMS over a domain-specific area (e.g. right PC – orientation) may then add to the inhibitory influence on that area from regions specialised for processing the other type of information (e.g. left IFG – colour). This would disrupt performance in the dual task, which requires processing in both pathways. rTMS over a domain-specific area may not affect performance on the preferred single task, because the starting level of activity is higher. In support of this idea, there is evidence to suggest that the influence of TMS on task performance depends on neural activity states, with TMS preferentially affecting the least active neural populations during both online TMS [67,68] and theta burst TMS [69].

The results reported here are consistent with visual search studies, which have shown that TMS over right parietal cortex [70,71] and prefrontal cortex [36] selectively impairs search for conjunctions, but does not affect single feature search. Visual search and memory search are thought to depend on a common network of brain regions [72], therefore it is possible that the same neural mechanisms are involved in integrating information in both visual search and memory search.

In contrast to the results of the present study, and findings from visual search paradigms [36,70], other work has shown that TMS over dorsal areas, including right parietal cortex, interferes with WM for spatial information such as locations [30–32], whereas TMS over ventral areas, including ventral prefrontal cortex, interferes with WM for object information such as abstract patterns and faces [31,32]. Similarly, there is evidence to suggest that manipulation, without memory, shows similar effects. A number of studies have shown that right parietal cortex is involved in mental rotation [73,74] and TMS over this region has been found to impair mental rotation [75,76] and visuo-spatial imagery [41]. An explanation for the lack of TMS effects on the single tasks in the current study could be that other regions were able to take over processing. Indeed, studies combining TMS with functional brain imaging have shown that TMS over one cortical area can induce changes in neural activity over the whole brain [77–80]. This is consistent with the idea that neurons in prefrontal cortex are highly adaptable to different cognitive demands [81]. Such neural flexibility may result in successful performance on single tasks, but not on the conjunction task. That is, single features (colour or orientation) could be adequately processed in less-preferred regions, whereas successful integration of features in the conjunction task may require the input of specialised areas.

As already noted, performance on the single colour and orientation tasks may not have been impaired by TMS in the present study due to higher initial activation in domain-preferred regions. Therefore, it is possible that the intensity of stimulation was not high enough to impair single feature task performance in this paradigm. Indeed, previous studies have used stimulation intensities of between 90 and 130% of resting motor threshold [30–32], whereas the present study used a much lower stimulation intensity (80% of active motor threshold) in order to keep within the safety recommendations for theta burst rTMS [82]. Furthermore, theta burst rTMS may suppress neural activity through different mechanisms than other types of TMS. For example, continuous theta burst rTMS is thought to produce long-term depression in excitatory synaptic connections [83].

In conclusion, we have demonstrated that offline rTMS over left inferior frontal and right parietal regions selectively impairs WM for colour-orientation conjunctions. Together with the fMRI results, this is consistent with the idea that domain-specific regions play a role in forming integrated object representations in WM. Cognitive coordination in tasks requiring integration of visual and spatial information may be mediated by functional coupling between parietal and frontal regions [66]. Our results have implications beyond the field of brain stimulation because the neurophysiological mechanisms highlighted here may also explain why patients with dementia are more susceptible than controls to dual task interference [84,85] and show impairments in processing feature conjunctions [86].

## Footnotes

1 A′ was calculated using the following formula: A′ = 0.5 + [(H – FA) × (1 + H – FA)]/[4 × H × (1 – FA)] where H is hit rate (proportion of correct responses on match trials) and FA is false alarm rate (proportion of mismatching trials incorrectly identified as matching). If FA > H the following formula is used: A′ = 0.5 – [(FA – H) × (1 + FA – H)]/[4 × FA × (1 – H)]. A′ of 0.5 indicates chance performance and A′ of 1 indicates perfect performance.
